# The impact of perception and presence on emotional reactions: a review of research in virtual reality

**DOI:** 10.3389/fpsyg.2015.00026

**Published:** 2015-01-30

**Authors:** Julia Diemer, Georg W. Alpers, Henrik M. Peperkorn, Youssef Shiban, Andreas Mühlberger

**Affiliations:** ^1^Clinical Psychology and Psychotherapy, Department of Psychology, University of RegensburgRegensburg, Germany; ^2^Chair of Clinical and Biological Psychology and Psychotherapy, Mannheim School of Social Sciences, University of MannheimMannheim, Germany; ^3^Department of Psychology I, University of WürzburgWürzburg, Germany

**Keywords:** virtual reality, perception, fear, anxiety, emotion, presence

## Abstract

Virtual reality (VR) has made its way into mainstream psychological research in the last two decades. This technology, with its unique ability to simulate complex, real situations and contexts, offers researchers unprecedented opportunities to investigate human behavior in well controlled designs in the laboratory. One important application of VR is the investigation of pathological processes in mental disorders, especially anxiety disorders. Research on the processes underlying threat perception, fear, and exposure therapy has shed light on more general aspects of the relation between perception and emotion. Being by its nature virtual, i.e., simulation of reality, VR strongly relies on the adequate selection of specific perceptual cues to activate emotions. Emotional experiences in turn are related to presence, another important concept in VR, which describes the user’s sense of being in a VR environment. This paper summarizes current research into perception of fear cues, emotion, and presence, aiming at the identification of the most relevant aspects of emotional experience in VR and their mutual relations. A special focus lies on a series of recent experiments designed to test the relative contribution of perception and conceptual information on fear in VR. This strand of research capitalizes on the dissociation between perception (bottom–up input) and conceptual information (top-down input) that is possible in VR. Further, we review the factors that have so far been recognized to influence presence, with emotions (e.g., fear) being the most relevant in the context of clinical psychology. Recent research has highlighted the mutual influence of presence and fear in VR, but has also traced the limits of our current understanding of this relationship. In this paper, the crucial role of perception on eliciting emotional reactions is highlighted, and the role of arousal as a basic dimension of emotional experience is discussed. An interoceptive attribution model of presence is suggested as a first step toward an integrative framework for emotion research in VR. Gaps in the current literature and future directions are outlined.

## INTRODUCTION

In virtual reality (VR), researchers can simulate intricate real-life situations and contexts to investigate complex human behaviors in highly controlled designs in a laboratory setting. These characteristics of VR have proven especially attractive for the investigation of pathological processes in mental disorders, and this technology has steadily gained momentum since the 1990s (Rothbaum, 2009). The main application of VR scenarios in this field is research into the processes underlying anxiety disorders and their treatment. Here, VR has become established as a medium for investigating threat perception, fear, and exposure treatment (Mühlberger et al., 2007; Rothbaum, 2009; Opris et al., 2012; Glotzbach-Schoon et al., 2013; Shiban et al., 2013; Diemer et al., 2014).

For research into emotional experiences and emotional behavior, such as fear, anxiety, and exposure effects, it is vital that VR can actually induce emotional reactions. By its very nature, VR as a medium is “unreal” and relies on perceptual stimulation (including perceptual feedback of one’s own actions) – in particular, visual cues, sounds, and sometimes touch and smell – to trigger emotional reactions. Historically, the first VR scenarios applied in the field of mental disorders used powerful visual stimuli to provoke emotional responses, in particular, height (Hodges et al., 1995). Soon, more complex multimodal presentations of visual, acoustic, and vestibular stimuli were developed, for example, to simulate airplane travel (e.g., Mühlberger et al., 2001, 2003, 2006). Still, as it is the very nature of VR the emotional cues relied on perceptional simulations. However, more recent studies have highlighted the need to consider not only bottom-up processes of perception, but also top-down effects when it comes to understanding how VR can be emotionally engaging – e.g., a background narrative to a VR scenario may enhance emotional experience (Bouchard et al., 2008; Gorini et al., 2011; Mühlberger et al., 2012; Peperkorn and Mühlberger, 2013). What is interesting about this perspective is that VR, as a perceptual medium (e.g., all experiences may be interpreted as not-evidence based), enables researchers to dissociate perceptual, i.e., bottom–up input, and higher-level, i.e., top–down processes based on information, and to manipulate them independently to study their effects separately and in combination.

Another VR phenomenon linked to emotional experience is presence. Presence is a dimensional construct and describes the extent to which a user feels present in a VR environment (Slater and Wilbur, 1997; Schubert et al., 2001; Botella et al., 2009). Theories of presence can be divided into descriptive and structural models. Descriptive models focus on delineating the components of presence, like the model embedded in the Igroup Presence Questionnaire (Schubert et al., 2001). Via factor analysis, these authors identified three dimensions of presence: spatial presence, involvement, and realness (Schubert et al., 2001). On the other hand, structural models aim at an understanding of how the experience of presence is generated in the mind. These models focus on cognitive processes and generally suppose that directing attention to the VR environment (e.g., Witmer and Singer, 1998) and creating a mental representation of this environment (Sheridan, 1999) are necessary processes that enable us to experience presence (Sheridan, 1999; Schuemie et al., 2001). The most recent structural model of presence, proposed by Seth et al. (2012), goes beyond earlier theories. Their perspective is not limited to VR, but instead, Seth et al. (2012, p. 12) point out that presence is an everyday phenomenon, “a basic property of normal conscious experience”. Seth et al. (2012) argue that extremes of disturbed presence (with regard to normal reality) can be observed, for example, in schizophrenia and depersonalization disorder. The basic precept of Seth et al.’s (2012) *interoceptive predictive coding model* is that presence rests on continuous prediction of emotional (interoceptive) states. For example, when expecting the encounter with an anxiety-related stimulus, the prediction would be fear, together with the changes the organism usually undergoes during fear. When encountering the feared stimulus, the organism compares the actual interoceptive state (fear and its symptoms) with the predicted state. According to Seth et al. (2012), there will practically always be a certain degree of mismatch. Seth et al. (2012) postulate that presence is the result of successful suppression of this mismatch between the predicted and the actual interoceptive state – i.e., the prediction prevails over the mismatch signals. The idea that suppression of information that is incompatible with the VR experience is vital for presence is not new (Schuemie et al., 2001). For example, Sheridan (1999) posits in his estimation theory that presence is the result of a continuously updated interior model of the environment, stressing the necessity for suspension of disbelief. However, Sheridan (1999) is concerned with the prediction of environmental, i.e., external events. What is unique to Seth et al. (2012) is their emphasis on the prediction of interoceptive states (rather than external events), which affords a crucial role to emotional experience.

The aim of this paper is twofold. First, we provide a review of current research into the relationship between perception and information on emotional experience in VR environments. Since exposure therapy has so far been the most common application of VR technology in clinical psychology, our focus lies on VR concerned with fear and anxiety in both healthy and clinical populations. We present a series of our own experiments that were designed to examine the significance of perception vs. conceptual information and presence for the experience of anxiety, and fear in VR environments. Second, an integration of the literature regarding immersion, presence, and emotional experience in VR is still outstanding. Different VR systems, diverging operationalizations of presence, and study samples ranging from healthy controls to patients with anxiety disorders make it difficult to draw firm conclusions. Based on a review of presence research, we suggest a new interoceptive attribution model of presence as a step toward an integrative framework for emotion research in VR.

## EFFECTS OF PERCEPTION VS. INFORMATION ON FEAR

The most influential theoretical conceptualization of dysfunctional fear to date is offered by the *emotional processing theory* by Foa and Kozak (1986; McNally, 2007). According to this theory, dysfunctional fear can be viewed as a memory network comprising information about the feared stimulus (e.g., its characteristics), the fear response (i.e., behavioral plans concerning escape and avoidance), and propositions of meaning (e.g., association with danger or threat; Foa and Kozak, 1986). Importantly, this fear network can be partly or fully activated by input that matches part of the network. Fear, according to this theory, is an index of network activation and can be measured both subjectively and physiologically (Foa and Kozak, 1986).

Fear can be activated by at least two pathways: The perceptual (e.g., visual fear-related cues) and the conceptual (fear-related information) paths. Perceptual fear-related cues are assumed to rapidly evoke physiological and behavioral fear reactions, whereas fear-related information is expected to produce subjective fear reactions, but only a poorer physiological activation (Hofmann et al., 2008). Strack and Deutsch (2004) in their *reflective-impulsive model of social behavior* propose that impulsive, emotional reactions are fast, and governed by the laws of association (spreading activation), while reflective behavior is subject to more flexible, cognitive control. However, the impulsive and the reflective systems are supposed to interact, allowing conceptual information (input to the reflective system) to activate rapid emotional reactions (Strack and Deutsch, 2004). In practice the separation of the two paths is difficult to investigate as emotionally relevant situations typically comprise input to both paths simultaneously.

Virtual reality is a particularly suitable tool as it offers an opportunity to differentiate the two paths for eliciting emotion. In VR, cue propositions can be activated by presenting feared objects perceptually (e.g., visually), and, unrelated to the perceptual presentation, activating the meaning propositions by informing a person of the existence of a feared object, or situation outside the VR scenario they are immersed in. The laboratory setting of VR further allows the online assessment of different fear reactions (subjective, physiological, and behavioral) in a highly controlled setting.

### EMPIRICAL FINDINGS ON SPECIFIC PHOBIA

In a series of studies we investigated the relative importance of perceptual fear-related cues and conceptual fear-related information on the activation of fear in different anxiety disorders. We assumed that fear reactions in specific phobia (animal type) are primarily caused by simple perceptual fear-related cues like a spider, whereas the impact of information on fear (i.e., knowing about the presence of a spider without seeing it) should be less pronounced. We directly and separately manipulated the two paths by using VR to present the visual cues on the one hand and the independent information about the existence of a real fear-evoking stimulus on the other hand.

**FIGURE 1:**
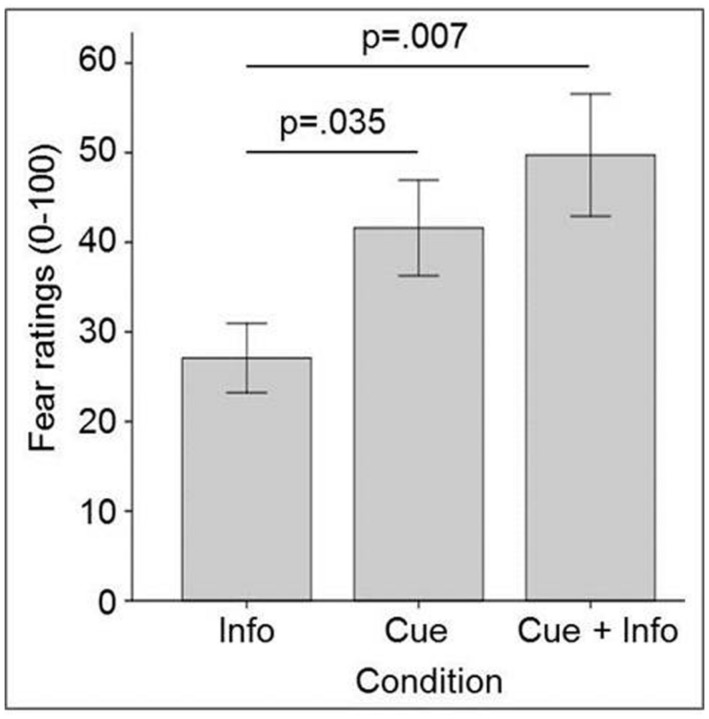
**Fear in spider phobia during exposure to spider cues in virtual reality (cue), to a real (but unseen) spider (info), and to both.** Error bars represent SEM.

In a first study with patients suffering from spider phobia (Peperkorn et al., 2014), we found that specific perceptual cues (in this case visual simulations of a spider) and conceptual information (verbal report that an unseen spider was present in front of the participant) presented separately activated the fear network, albeit via different routes. Specifically, perceptual cues vs. conceptual information led to different degrees of fear activation, with the perceptual route being significantly more fear provoking than the informational route, as was expected for spider phobia. Fear ratings (mean of five exposure trials) of this experiment are shown in **Figure 1**.

In a second study, we addressed the question whether these findings generalize to other types of phobias. While in spider phobia, fear is characteristically triggered by a stereotypical object (the animal), in other phobias – those of the situational subtype, e.g., claustrophobia – triggers are more context-related, involving more complex perceptual stimuli (American Psychiatric Association, 2013). Therefore, we used the same design in a sample of patients suffering from claustrophobia (Shiban et al., submitted). Similar to the spider phobia study (Peperkorn et al., 2014), we found for claustrophobia that the perceptual condition (seeing the inside of a virtual box with a closed door) initially activates stronger self-reported and physiological fear responses compared to the information condition where patients knew they sat in an actual, closed claustrophobic box (the fear-specific information), but saw an open door in the corresponding VR environment. It is important to note that although both studies used mainly visual cues as perceptual cues, in the spider phobia study the cues were specific (a virtual spider), whereas in the claustrophobia study they were more complex and context-related (a claustrophobic box).

In summary, in these studies we demonstrated for the first time in an integrated multimodal experiment that perceptual cues and conceptual information can provoke fear reactions in specific phobia, with additive effects if combined. Interestingly, perceptual cues alone seem to induce more self-reported fear than information alone, regardless of the type of specific phobia (animal vs. situational subtype). This is in line with findings that fear enhances perceptual, but not mental processing (e.g., Borst and Kosslyn, 2010), implying that there is a closer link between perceptual input and the experience of fear, than between fear and the mental processing of (purely conceptual) information.

### EMPIRICAL FINDINGS FOR SOCIAL ANXIETY

As social fears are generally thought to be more cognitive in nature than specific phobias (Clark, 2005; Schulz et al., 2008; Wieser et al., 2010), we expected – in contrast to the results from studies on specific phobia – that anticipating a speech would be more fear-provoking when conducted in front of an audience a participant is informed are there (even if not seeing the audience: information condition) than in front of a virtual audience (perceptual cues) when knowing that actually no one will listen to the talk. Therefore, in a third study we applied a modified version of the paradigm described above to a public speaking challenge (Shiban et al., 2014; Diemer et al., in preparation). In contrast to the studies of specific phobia, anticipatory anxiety was chosen to avoid a possible confound in the physiological variables due to arousal caused by speaking (Gramann and Schandry, 2009). Also, anticipatory anxiety has been shown to share important parts of the neural network of acute anxiety (Nitschke et al., 2006).

**FIGURE 2:**
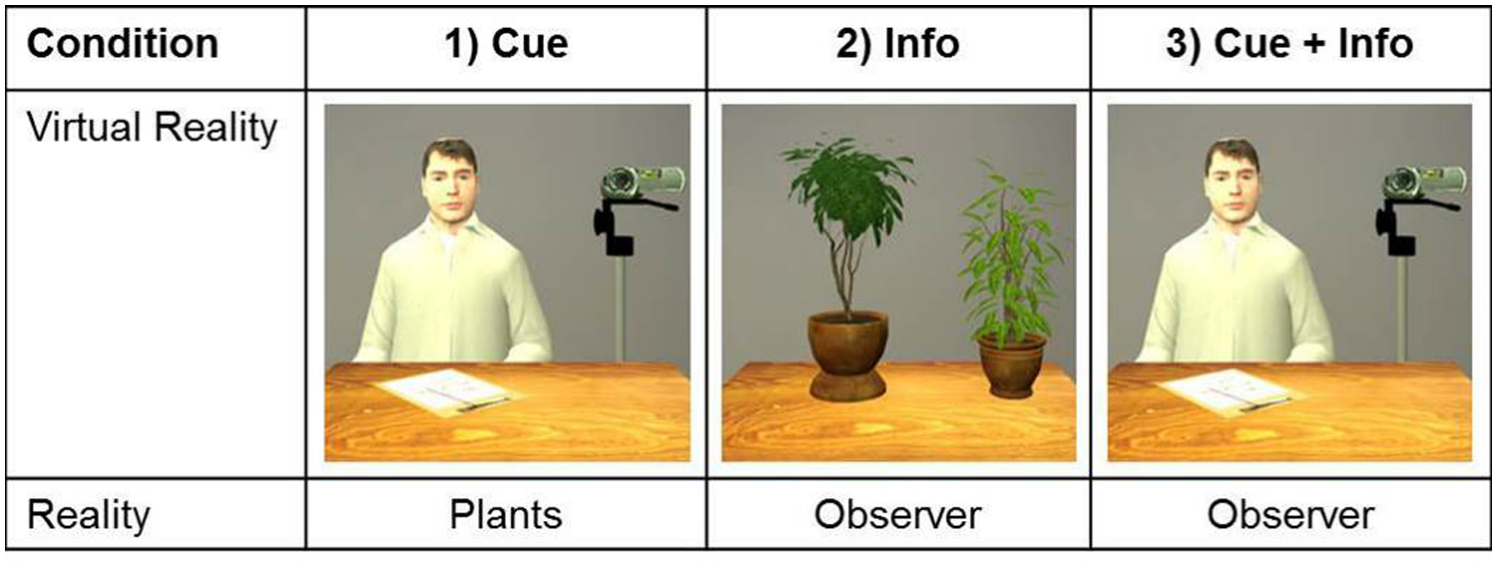
**Manipulation of fear cues and fear-relevant information in public speaking**.

**FIGURE 3:**
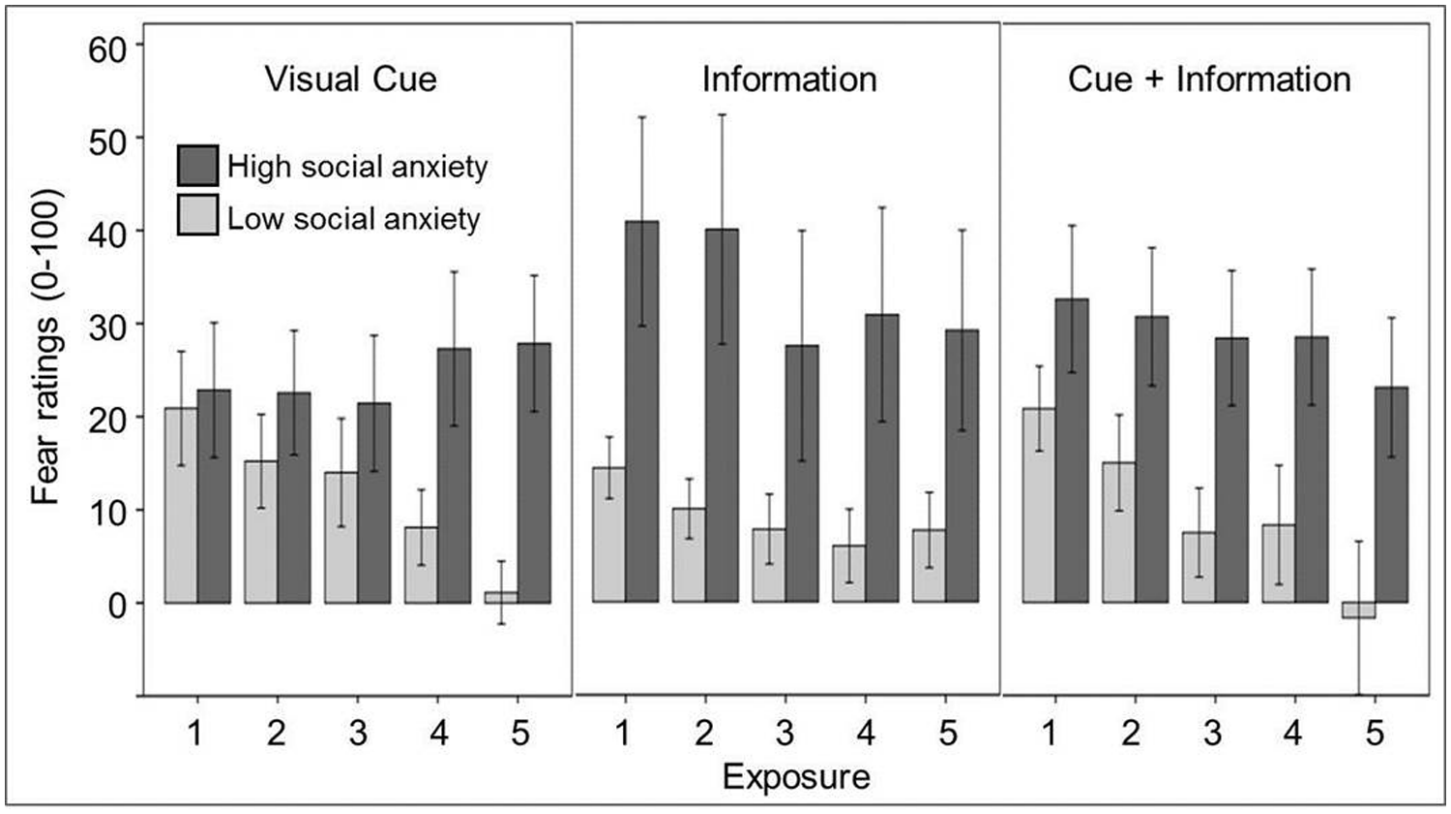
**Anticipatory anxiety of high and low socially anxious participants. Difference scores to baseline are given.** Error bars represent SEM.

We hypothesized that a real observer outside VR (information condition) would evoke significantly stronger subjective and physiological fear reactions than a visual observer in VR (perceptual cue condition). Further, we expected that a combination of real and VR audience (combined condition) would result in the strongest subjective and physiological fear reactions. The experimental conditions are presented in **Figure 2**. Finally, we expected high socially anxious participants to show stronger fear reactions than low socially anxious participants. We randomly allocated 48 healthy participants to either the information condition, the cue condition, or the combined condition. (for details of physiological data acquisition, see Peperkorn et al., 2014). As expected, socially anxious participants reported significantly higher subjective fear, but there were no differences between conditions (see **Figure 3**). Physiological parameters [heart rate, skin conductance level (SCL)] decreased significantly over time. There was a trend SCL to differ between groups, with the highest SCL in the visual cue condition (*p* = 0.066), but there were no other effects of social anxiety or condition. With a mean Social Phobia Inventory (SPIN; Connor et al., 2000) score of 21.8 (median: 21, SD: 10.5), our sample was above the mean of healthy controls (*M* = 12.1, SD = 9.3), but markedly below the mean (*M* = 41.1, SD = 10.2) of patients with social phobia reported by Connor et al. (2000). While these results are disappointing in sofar as we could not find the expected effect of the information condition, the paradigm has shown promise. There was a clear effect of social anxiety, with significantly higher subjective fear in socially anxious participants, and in contrast to the studies on specific phobia, no superiority of the cue condition was found (Shiban et al., 2014; Diemer et al., in preparation). Therefore, we believe that it would be worthwhile to apply this paradigm in a larger sample of patients with social anxiety disorder, and to assess acute fear during public speaking.

### SUMMARY

In summary, the VR designs reported here confirmed the possibility of eliciting fear reactions via different routes (perceptual vs. conceptual). Patients with specific phobia seem to be particularly sensitive to perceptual cues. Interestingly, this finding was the same for spider phobia (animal type) and claustrophobia (situation type). For social anxiety, no differences in activation of the fear structure between the two paths were found. These observations are in line with Foa and Kozak’s (1986) prediction about differential sensitivities of different anxiety disorders to different media of exposure (*in vivo* cues vs. imagination). However, the interpretation of our results on social anxiety remains preliminary, as we did not assess patients or acute fear as in the studies of specific phobia. It seems worthwhile to continue this research with different kinds of specific phobias and more complex anxiety disorders like agoraphobia, panic disorder, and social anxiety disorder.

## PRESENCE AND EMOTION IN VR

The association of presence and emotional experience in VR exposure therapy is an issue of debate. Presence is commonly regarded as a necessary mediator that allows real emotions to be activated by a virtual environment (Parsons and Rizzo, 2008; Price et al., 2011). While this conception implies a causal role for presence, research has not yet been able to clarify the relationship between presence and emotional experience in VR.

Presence has been conceptualized, and consequently operationalized and manipulated, in very different ways. These ranges from a manipulation of presence by providing more or less sophisticated VR technology to the diverse methods of assessing presence, either by subjective ratings taken online during the VR experience, or afterward via questionnaires. Presence questionnaires vary greatly with regard to the constructs they measure; however, what they have in common is that they ask participants for a subjective judgment regarding their experience of presence. With this in mind, we will use the definition of Slater and Wilbur (1997) and Slater (1999) and call any manipulation at the level of technology a manipulation of immersion, rather than presence. Presence is defined as a subjective phenomenon that results from experiences induced by immersive VR technology (Slater and Wilbur, 1997; Slater, 1999; Schubert et al., 2001). To avoid confusion with aspects of immersion (technology), for the purpose of this paper, only subjective measures of the presence experience (ratings or questionnaires) are considered presence measures. We chose not to include physiological parameters as indicators of presence as physiology is directly linked to emotional arousal, so considering physiological responses as operationalizations of presence would inevitably bring a confound of presence and emotion. The following section on presence and emotion considers two approaches to presence. First, the effects of immersive VR technology on presence and emotion are considered. Then, we will take a closer look at correlative findings of presence and emotion.

### THE ROLE OF IMMERSION

#### Immersion and presence

VR simulations can be more or less graphically enhanced, multimodally integrated, and interactive. More sophisticated technology is often thought to result in more presence. Already Botella et al. (1999) reported more emotional reactions to a simple, neutral VR scene when a high-quality head-mounted display (HMD) was used, compared to a medium-quality HMD. Typically, studies assessing different degrees of immersion find higher presence in more immersive VR systems compared to less sophisticated setups. Such effects have been reported for VR scenarios presented via a Cave Automatic Virtual Environment (CAVE) vs. HMD (Krijn et al., 2004; Juan and Perez, 2009), for HMD vs. computer monitor (Gorini et al., 2011), video wall (a large stereoscopic projection screen) vs. computer monitor (Baños et al., 2004), for active vs. passive navigation in VR (Freeman et al., 2005), and for stereoscopy vs. monoscopy (Ijsselsteijn et al., 2001; Ling et al., 2012). Although some researchers have failed to find an effect of immersion on presence (e.g., Baños et al., 2008, for stereoscopy), in general, research indicates that more sophisticated simulations (higher immersion) result in increased presence, especially in virtual environments not designed to induce particular emotions (Baños et al., 2004).

#### Immersion and emotion

As for possible effects of immersion on emotions, the picture becomes more complicated. While some authors report an increase in emotional responses in more immersive compared to less immersive VR systems (Botella et al., 1999; Juan and Perez, 2009; Visch et al., 2010), others did not find effects of immersion on emotion (Freeman et al., 2005; Ling et al., 2012). In more detail, it seems that immersion effects on emotion might depend on the nature of the emotions under study. Visch et al. (2010) suggest that the effect of immersive technology is mediated by arousal. This idea appears plausible, as especially fear and anxiety, both of which are strongly arousing emotions, have been found to be stronger in more immersive VR setups (Juan and Perez, 2009), while happiness and relaxation appear to be much less influenced by the technology used (Freeman et al., 2005; Baños et al., 2008). Of note, the positive emotions induced in the studies by Freeman et al. (2005) and Baños et al. (2008) were not only of different valence than fear, but also non-arousing in nature. In a study of spider phobia, we also found stronger subjective and behavioral (avoidance) fear reactions in a stereoscopic vs. monoscopic VR (Peperkorn et al., submitted). By contrast, Ling et al. (2012) did not find an effect of stereoscopy on emotional reactions including fear. However, Ling et al. (2012) investigated healthy participants during a speech task, so arousal levels (mean heart rate about 75 beats per minute) appear to have been comparatively low.

Another possibility to test the influence of immersion is the use of different perceptual modalities or multimodal perceptual cues. Thus, we compared tactile cues (touching a spider model) with visual cues (visual VR spiders presented in the HMD) in patients with spider phobia (Peperkorn and Mühlberger, 2013). As expected, the combination of visual and tactile cues led to the highest fear ratings. Tactile cues alone activated significantly stronger fear reactions than visual cues alone. Interestingly, presence was also higher in the multimodal (perceptual plus tactile cues) than the single modus conditions, a finding that confirms the association of immersion and presence. However, the different perceptual paths that we investigated are few out of many; for example, acoustic stimuli can be important in specific phobia, and can be easily implemented in VR (Taffou et al., 2012).

Taken together, there is considerable evidence that the level of immersion a VR system provides exerts an effect on the presence experienced by the user (Ijsselsteijn et al., 2001; Freeman et al., 2005). This effect seems to be particularly prominent in the absence of emotional manipulations, i.e., the effect does not seem to be mediated by emotion. The fact that immersion does not *per se* increase emotional experience, but that the emotionally enhancing effect of immersion might be limited to arousing emotions (see the discussion above), supports this conclusion. For example, Baños et al. (2004) independently manipulated immersion (HMD vs. computer monitor vs. video wall) and emotional content (sad vs. neutral) of a VR scenario. They found an interaction effect, with immersion affecting presence ratings in the emotionally neutral condition, but much less so in the emotional (sad) condition. There was also a main effect of emotion, with higher presence in the emotional than in the neutral condition (Baños et al., 2004). However, it is not clear from these data why there was no immersion effect on presence in the emotional condition; unfortunately, Baños et al. (2004) do not report the strength of the actual emotions experienced by their participants. As manipulations of immersion are not direct manipulations of presence, it is impossible to determine from these findings whether presence is causal for emotional experience. It has been argued that immersion causes arousal, which in turn increases presence and emotion ratings (Visch et al., 2010). We will come back to the issue of arousal in the following section on correlative findings.

### PRESENCE AND EMOTION

The association of presence and emotion has been mainly investigated by means of correlations between these two measures. Correlations between presence and emotional experience in VR have been consistently reported, especially in the literature on VR exposure therapy (Robillard et al., 2003; Price and Anderson, 2007; Riva et al., 2007; Bouchard et al., 2008; Alsina-Jurnet et al., 2011; Price et al., 2011), although some researchers have reported no relation between presence and the extent of experienced fear (Krijn et al., 2004). A common conclusion in this type of research is that in VR exposure therapy, presence and fear appear mutually dependent (Robillard et al., 2003; Price and Anderson, 2007). In a recent study, we confirmed the positive association, but additionally found indications that the relationship between presence and fear might change dynamically during exposure to phobic stimuli (Peperkorn et al., submitted). Interestingly, a general effect of presence on treatment outcome could not be established (Krijn et al., 2004; Price and Anderson, 2007), although Price et al. (2011) found that scores on the presence subscale “involvement,” but not other presence scales, predicted treatment outcome in a sample of patients with social phobia (*n* = 31) undergoing VR exposure therapy.

In the case of fear in non-patients, results are less clear. On the one hand, there are results paralleling findings from patient samples. For example, Alsina-Jurnet et al. (2011) exposed a large sample (*n* = 210) of test-anxious students and non-anxious students (groups assigned according to questionnaire scores) to a VR environment that simulated a university exam, and a neutral VR. The authors reported no correlation between fear and presence in the neutral VR, and a considerably stronger correlation between presence and fear in the test anxious group (Alsina-Jurnet et al., 2011). We found similar results in a sample of spider fearful and control participants exposed to VR spiders, with significantly stronger presence in the fearful participants vs. controls, and a significant positive correlation between presence and fear in the fearful participants only (Peperkorn and Mühlberger, 2013). Whether this pattern of results is related to a floor effect and/or reduced variability in fear ratings in the healthy samples has not been investigated. On the other hand, research on emotions other than fear tends to produce mixed results. In an emotion induction paradigm in VR, Baños et al. (2004, 2008, 2012) tested the effects of different kinds of emotion on presence. They found correlations between presence and emotion in healthy controls for sadness (Baños et al., 2004), joy (Baños et al., 2008), and relaxation (Baños et al., 2008). Using non-immersive VR equipment, Baños et al. (2012) could not find significant correlations between emotion (joy, relaxation) and presence; however, they did observe relatively high presence ratings. By contrast, using a relaxation paradigm presented with different levels of immersion, Freeman et al. (2005) found only one significant correlation between the experience of happiness and a presence scale, which the authors interpreted as an artifact due to item overlap.

Interestingly, some authors have also tested the effects of emotions induced by information on presence. Gorini et al. (2011) had participants search for a blood container in a VR hospital, either with the information that this was urgently needed to save a child, or without this information. Bouchard et al. (2008) informed patients with snake phobia that there were snakes in a VR environment, while in fact, no snakes were shown. Both Bouchard et al. (2008) and Gorini et al. (2011) reported that this emotionally relevant background information enhanced presence, indicating a causal influence of emotions on presence. Other possible influences on presence could be personality or (spatial) intelligence (Alsina-Jurnet and Gutierrez-Maldonado, 2010). However, little is yet known about the influence of these, or other, traits on presence or emotion during a VR experience.

Taken together, results show that the stronger the feelings involved, either because of the nature of the emotion (e.g., fear vs. joy vs. relaxation), or because of the nature of the sample (patients with anxiety disorder vs. normal controls), the greater the likelihood of finding a significant correlation between presence and emotion. A possible explanation for this phenomenon could again be arousal. Already, Freeman et al. (2005) suggested that the correlation of presence and emotion might be limited to arousing stimuli. They proposed an arousal theory of presence, arguing that arousal leads to alertness, which in turn leads to higher presence ratings. According to Freeman et al. (2005, p. 2018), alertness increases a participant’s readiness to respond to the stimuli that compose a given VR, as arousal represents a “call to action” – thus leading to a greater perceived physical and mental presence in VR. So far, this arousal theory has not been rigorously tested, although objective measures of arousal (i.e., physiological parameters) can be easily assessed during VR sessions (Mühlberger et al., 2007; Diemer et al., 2014). First evidence for a crucial role of arousal comes from the study by Gorini et al. (2011), who reported significantly higher heart rate in the group that experienced the hospital VR with a narrative that increased the relevance of the scenario. Unfortunately, Gorini et al. (2011) do not report correlations between heart rate and presence ratings.

## DISCUSSION

The findings reviewed here highlight important advances in the study of fear and anxiety in VR environments. The data on perceptual fear cues and conceptual information show that both are viable triggers of fear reactions (Bouchard et al., 2008; Gorini et al., 2011; Peperkorn et al., 2014; Shiban et al., in preparation). There is evidence that patients with specific phobia react more strongly to visual cues than to fear-specific information, a finding that lends preliminary support to dual-process theories like the impulsive–reflective model of social behavior (Strack and Deutsch, 2004). The possibility of activating fear separately by perceptual cue or information in VR opens up new research opportunities to investigate pathological processes specific to each route. This might be particularly relevant for cue-independent fears and anxiety, for example in obsessive–compulsive disorder, illness anxiety disorder, and generalized anxiety disorder.

As for presence, the literature shows the significance of immersion on presence. Specifically, greater immersion of a VR system increases presence, particularly in emotionally neutral VR scenarios, which indicates that the effect is not mediated by emotion. In fact, it seems that the “depth” of a VR experience in terms of presence and emotion is more strongly influenced by factors quite apart from the technological quality of the VR system. Certainly the effect of immersion – i.e., technological quality – on presence exists, but interestingly, it is strongest when no emotion is involved. As soon as a VR scenario engages emotions, presence is increased. Studies that manipulate emotion independently of the technological aspects and even the stimuli presented via VR (e.g., Gorini et al., 2011) demonstrate this effect quite convincingly. Further, correlations between (strong) emotions and presence have been consistently reported. The effects of immersion and emotion on presence are possibly explained by arousal (Freeman et al., 2005; Visch et al., 2010), but theories of emotion and presence in VR (Freeman et al., 2005; Seth et al., 2012) have so far been insufficiently tested. In the case of VR exposure therapy, neither general presence nor immersion seem to be related to treatment outcome (Mühlberger et al., 2005); rather, a certain degree of both appears a necessary requirement for VR exposure therapy, but increasing either does not *per se* enhance therapy effects (Krijn et al., 2004; Price and Anderson, 2007).

Before the findings reported here can be integrated into one model, more research is needed. While the data resumed so far indicate a crucial role for arousal, the position of arousal in an explanatory framework that comprises VR system factors, immersion, aspects of stimulation (e.g., perception vs. information), presence and emotion is not clear. First, we do not know how the effect of perception vs. information on emotion is produced. On the one hand, fear-related elements in VR are input cues to the fear network – as proposed in emotional processing theory (Foa and Kozak, 1986) – and might thus directly enhance emotional arousal. However, this theory does not explain why, in specific phobia, perceptual cues have a stronger effect on fear network activation than information alone. The reflective–impulsive model of social behavior (Strack and Deutsch, 2004) can explain different effects of perception vs. information on fear. On the other hand, however, emotionally relevant perceptual stimuli and information enhance a VR environment, making it more interesting, appealing to attention and ultimately, increasing, at least initially, arousal – irrespective of the emotional valence of the stimuli in question. Since arousal is a basic dimension of emotional experience, the effect of perception and information on emotion might be mediated by arousal. The role of arousal should be tested with emotions with different levels of arousal, using in particular physiological indicators of arousal. To broaden the range of emotions investigated, anger would be interesting as a highly arousing emotion other than fear that could also be activated in VR.

**FIGURE 4:**
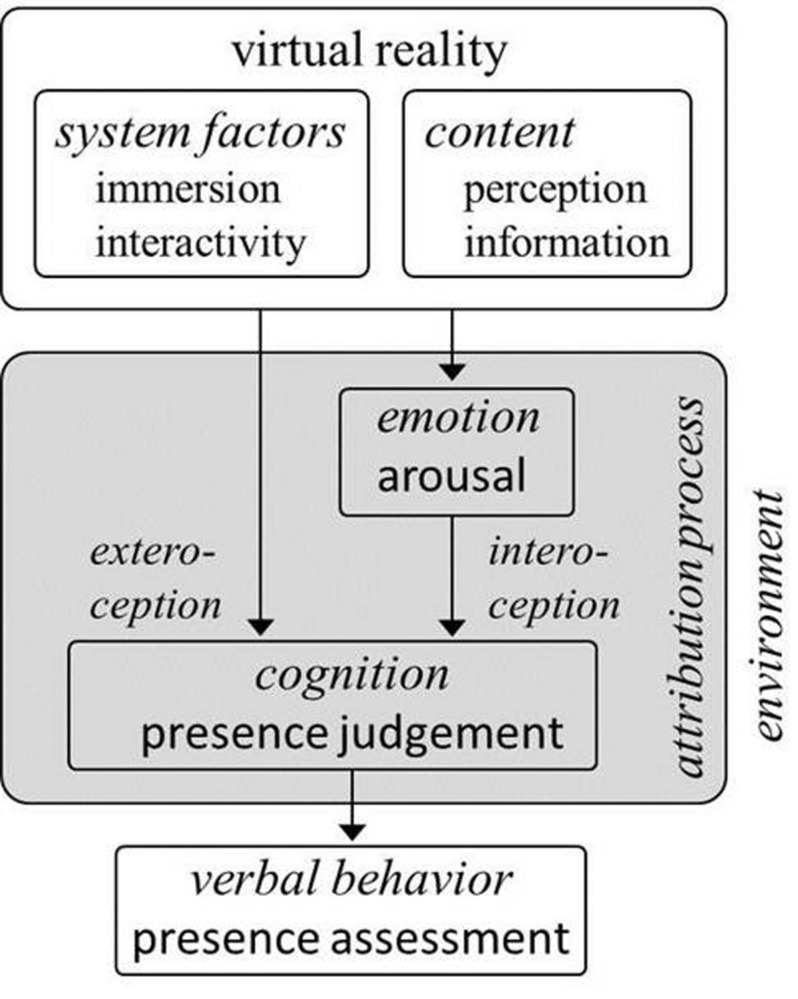
**An interoceptive attribution model of presence**.

Concerning presence, the preliminary conclusion we would draw from the findings reviewed here is that the case for a crucial involvement of arousal in the experience of presence is compelling. However, the mechanism of this effect cannot be discerned yet. Freeman et al. (2005) propose that arousal increases presence by enhancing attention to a VR environment and the possibilities of action offered by this environment. A different explanation we suggest is an *interoceptive attribution model of presence* (see **Figure 4**). Since presence is a subjective experience, common measures of presence explicitly call the participants to make a judgment of the degree of presence they feel in VR. Based on the results reviewed in this paper, we propose that participants make this judgment based mainly on two sources of information: (1) immersion and (2) the degree of arousal they feel. As for immersion, participants might base their presence judgment on the perceptual distance they experience from the real world setting, i.e., the less stimulation they receive from the real world, and the more stimulation from the VR scenario, the higher the level of presence they will indicate. Of course, this hypothesis needs further empirical confirmation. With regard to emotion, we believe that participants will give higher presence ratings if they feel emotionally affected. As arousal is a particularly strong indicator of emotional involvement, arousing emotions should lead to higher presence ratings, and correlate more closely, with presence ratings, than calm or serene emotional states – a picture that is in fact found in the literature. Interestingly, whether the experience of arousal *per se*, or the attribution of this arousal to the VR scenario is necessary for the experience of presence has not yet been investigated. Additionally, immersion itself is likely to increase arousal (Visch et al., 2010). In essence, the cognitive nature of presence – in that it is a subjective judgment – forms the core of our understanding of presence as it is usually conceptualized and assessed in its relation to immersion, stimulation, and emotion in VR research. We believe that our model is compatible with the predictive coding mechanisms put forward by Seth et al. (2012). In contrast to Seth et al.’s (2012) conception, our model focuses on the attribution process that gives rise to cognitive presence judgments. It is intended as a framework for research into emotional experience and presence in VR. Future studies should therefore differentiate as precisely as possible between cognitive presence (presence as a subjective judgment), emotional presence (Seth et al., 2012), and on the other hand immersion (technological features of a given VR system), arousal (as a dimension of emotion), specific emotions (along both the arousal and the valence dimensions), and the population under study (patients vs. fearful participants vs. healthy controls). Further, to fully understand presence in VR and its unique characteristics, the investigation of presence in reality, e.g., during *in vivo* exposure as compared to VR exposure, appears vital (Seth et al., 2012). We can reasonably assume that, when making sense of a VR environment, people apply the same mechanisms to it as they do to everyday reality (Seth et al., 2012). A direct comparison of both worlds has, unfortunately, long been neglected.

## AUTHORS CONTRIBUTION

Julia Diemer: data analysis, wrote the manuscript. Georg W. Alpers: study conception, contribution to data analysis, and the manuscript. Henrik M. Peperkorn: study conception, data acquisition, and analysis, contribution to the manuscript. Youssef Shiban: data acquisition and analysis, contribution to the manuscript. Andreas Mühlberger: study conception, data analysis, contribution to the manuscript. All authors have approved of the final version of the manuscript and its submission.

## Conflict of Interest Statement

The Guest Associate Editor Fritz Strack declares that, despite being affiliated to the same institution as author Henrik M. Peperkorn, the review process was handled objectively and no conflict of interest exists. Andreas Mühlberger is stakeholder of a commercial company that develops virtual environment research systems. Julia Diemer, Georg W. Alpers, Henrik M. Peperkorn and Youssef Shiban have no conflicts of interest.
